# Calibration of Off-the-Shelf Anisotropic Magnetoresistance Magnetometers

**DOI:** 10.3390/s19081850

**Published:** 2019-04-18

**Authors:** Leonard Schulz, Philip Heinisch, Ingo Richter

**Affiliations:** Institute for Geophysics und Extraterrestrial Physics, Technische Universität Braunschweig, 38106 Braunschweig, Germany; p.heinisch@tu-bs.de (P.H.); i.richter@tu-bs.de (I.R.)

**Keywords:** anisotropic magnetoresistance magnetometer, calibration, digital sensor, magnetic laboratory, attitude determination, commercial of-the-shelf sensor, polynomial calibration model

## Abstract

Magnetometers based on the anisotropic magnetoresistive effect are used in many applications for orientation determination, by measuring the magnetic field of the Earth. As sensors of this type are commercial, off-the-shelf components, manufacturers provide limited information on their measurement performance. Therefore, we present a (to date) unprecedented comprehensive calibration study on three state-of-the-art digital anisotropic magnetoresistance magnetometers, to precisely determine various performance parameters and stability across different sensors of the same model. With the evaluation of sensitivity, noise, offset, and orientation determination, as well as considering dependencies on temperature and frequency, the performance of each sensor can be improved significantly, enabling their implementation in demanding fields of application (such as in satellites). Different measurement and calibration techniques, specifically aimed at the characteristics of the examined magnetometers, were utilized, using a sophisticated magnetic laboratory that has served as a calibration facility for several interplanetary space missions. Our study allows operators to decide whether to consider anisotropic magnetoresitance magnetometers for their application and, more importantly, to be able to (at least partially) skip a time-intensive and complicated calibration by using the sensor parameters given in this paper. To that end, the most promising sensor is recommended. The sensor examination suggests a good comparability of different sensors of the same model, and shows the importance of noise regarding the sensor performance with a noise floor up to 124 nT/$\sqrt{\mathrm{Hz}}$ at 1 Hz. Additionally, depending on the sensor model, the sensitivity is 14 nT at best, and the attitude determination error can be reduced to about 0.3° with the given calibration.

## 1. Introduction

Anisotropic magnetoresistive (AMR) magnetometers are used in a variety of applications for angle measuring, attitude determination, and even magnetic field observations for space science [1]. They cover the sophisticated fields of measurement, such as navigation systems, by supporting GPS measurements or drones for attitude determination [2,3,4]. Most notably, they are used in small satellites [5,6,7]—namely, Cubesats—because of their mechanical ruggedness, compactness, and temperature endurance. In most fields of application, AMR magnetometers serve as electronic compasses to determine orientation by measuring the Earth’s magnetic field in three (nearly) orthogonal components [8]. Especially in spacecraft, the exact knowledge of the attitude is crucial; for example, in antenna and solar panel alignment, but also for proposed optical linking [9]. Therefore, a precise calibration of the magnetic sensors is vital to the success of spacecraft missions or the proper functionality of an electronic device.

AMR magnetometers can be divided into analog and digital ones, which differ in sensor structure and data output: The former consist of a Wheatstone bridge of AMR material [10]. The magnetic field along one spatial direction in the plane of the Wheatstone bridge can be derived from the measured voltage. Digital AMR magnetometers, additionally, have an integrated circuit for analog to digital signal conversion. Additionally, they mostly consist of three Wheatstone bridges, in order to cover all spatial directions. They show a significantly lower resolution and higher noise than analog AMR magnetometers, due to their higher degree of integration. Nevertheless, they can make up for these disadvantages by being cheaper, easier to integrate, and by having internal compensation (e.g., for temperature effects).

The manufacturer’s specifications for digital AMR sensors are mostly insufficient for an accurate determination of the magnetic field, as a precise calibration is not carried out by the manufacturer. In this paper, three commercially-available high performance digital AMR magnetometers are examined and information about their typical behavior and performance is given. The sensor models were chosen based on a preceding, extensive market analysis regarding specified performance. A preliminary examination of the most promising sensor models was performed; namely, the MAG3110 by NXP Semiconductors, the MPU-9250 by TDK InvenSense, the MMC5883 by MEMSIC, the HMC5883 and the HMC5893 by Honeywell, and the LSM303DLHC and LIS3MDL by ST Microelectronics. The last three were selected for further evaluation in this paper. Several different sensors of the same model are analyzed in order to obtain information about comparability across the same sensor model. We report on calibration techniques which surpass the heading precision of other methods (e.g., those mentioned and introduced in [11]), accommodate for nonlinear sensor responses, and give precise information on important sensor parameters, such as sensitivity, noise, misalignment, and offset, as well as temperature and frequency dependencies. Those parameters can be used by operators to help their decision-making on whether AMR magnetometers are suitable for their application and, especially, to provide standard performance parameters for the examined magnetometer models. By this, a time-intensive and complicated calibration can be (at least partially) avoided. This is very useful when a sophisticated calibration facility is not available.

The paper is structured as follows: In Section 2, the calibration facility that was used to calibrate the magnetometers for various past and current solar system space missions, such as Cassini [12] or Rosetta/Philae [13], is presented. Additionally, the calibration models and evaluated sensors are introduced and the calibration process is described. Section 3 presents the findings of the study, giving a complete parameter table for all sensor models. Finally, the findings are concluded and the best sensor is recommended, based on the overall performance, in Section 4. Additionally, advice is given on how to use the presented parameters.

## 2. Materials and Methods

### 2.1. Calibration Facility

Since 1967, the magnetic laboratory Magnetsrode has hosted many research projects and, from the 1980s, has served as a calibration facility for various space missions carrying a magnetometer [14]. To present, more than 14 magnetometers for use in space missions (starting with Giotto) investigating the Earth’s magnetosphere and reaching out to other planets, asteroids, and comets have been calibrated and tested. The most recent are Hayabusa 2/Mascot [15], JUICE [16], BepiColombo [17], and SOSMAG, the European Space Agency’s AMR magnetometer project for space applications [7].

**Figure 1:**
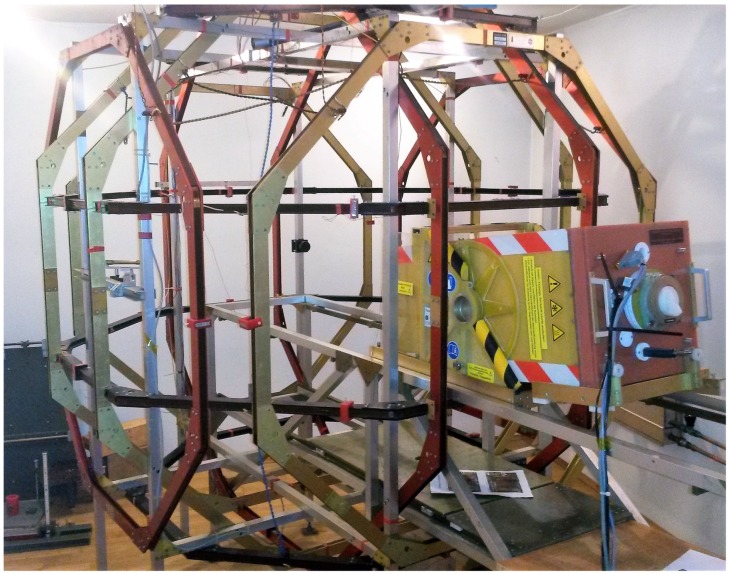
Braunbek coil system of the Magnetsrode calibration facility. The sensor under test is placed inside the yellow measurement box, which is then pushed into the middle of the coil system and set upright for the measurement.

The laboratory complex is comprised of four buildings and a three-axis fluxgate magnetometer, located in an underground bunker. In the latter, the ambient magnetic field is constantly measured in three components, with an absolute error below 1 nT. It is compensated with a Braunbek coil system [18] installed in the second building (see Figure 1). Thus, a quasi-zero field with an error in the order of 1 nT is achieved in the center of the coil system. The magnetic field range of up to 100,000 nT in each coil also enables every possible configuration of the Earth’s magnetic field to be reached, and even higher fields for industrial-type measurements.

The magnetic field sensor being tested is placed inside a thermally isolating box (yellow box in Figure 1), which is then aligned with the center of the coil system using a vertical laser beam. This setup allows temperature measurements in the range of −196 to 200 °C, with a built-in temperature control system. Additionally, it is possible to tilt and even rotate the sensor in the box. This setup also assures the comparability of all measurements taken. A detailed inside view of the measurement box is shown in Appendix A.

The Braunbek coil system is operated using computer-controlled devices to generate the desired artificial magnetic fields for all calibration tasks; namely, the determination of offsets, sensitivity, misalignment, and frequency response of the device under test.

All measurements, except the noise studies, reported here were carried out at Magnetsrode. The sensor noise was determined in a three-layer mu-metal shielded container, so external magnetic fields were not present during the measurement.

### 2.2. Calibration Models

For the comparison of calibration results, it is important to have standardized evaluation techniques. In this section, these techniques are introduced starting with the linear calibration model [14,19], which has been successfully used to calibrate various magnetometers for space missions [13]. Next, the polynomial calibration model, which is introduced here for digital AMR sensors—especially addressing their sensitivity behavior—is presented. In this paper, sensitivity is defined as a coefficient multiplied by the sensor output in order to obtain the corresponding magnetic field. Therefore, the unit of sensitivity is nT/LSB, and a lower sensitivity means better resolution of the magnetometer.

#### 2.2.1. The Linear Calibration Model

The relation between the calibrated applied magnetic coil field vector $B_{\mathrm{cal}}$ and the field vector measured by the sensor $B_{\mathrm{mea}}$ can be expressed by

(1) $$B_{\mathrm{cal}}^{T}=M\cdot \left(B_{\mathrm{mea}}-B_{\mathrm{off}}\right):=M\cdot B_{p},$$

where $B_{\mathrm{off}}$ is the sum of the sensor offset and the coil system residual field, and $B_{\mathrm{off}}$ is deducted from the sensor field vector, obtaining a new field vector $B_{p}$. Each measurement consists of many pairs of field vectors. The transfer matrix M can be acquired by solving the over-determined minimization problem given by the Euclidean norm

(2) $$\min{∥M\cdot B_{p}-B_{\mathrm{cal}}^{T}∥}_{2}^{2},$$

in the occasion of a trust-region algorithm, based on [20,21]. The transfer matrix can be separated into three matrices as

(3) M=R·W·S

where S and W contain information about the sensor’s sensitivity and internal misalignment, respectively. The rotation matrix R only depends on the orientation of the sensor’s base vectors with respect to the coil system’s base vectors. Therefore, this matrix is only dependent on the actual geometrical setup and can be disregarded for the sensor calibration matrix. A complete calibration matrix W·S, which is normally temperature and frequency dependent, is obtained for the sensor. The sensitivity matrix S is a diagonal matrix containing the sensitivity coefficients for each axis in sensor coordinates. They are gained by calculating the Euclidian norm of each column of the base-vector system transformation matrix. The misalignment matrix W is derived from the three misalignment angles of the sensor coordinate system, and is an upper triangular matrix. Knowing all three other matrices, R can be calculated, and the three Euler angles determining the sensor rotation, with respect to the coil system, are obtained. With the knowledge of the nine independent elements of M, all six angles and three sensitivities can be determined uniquely.

#### 2.2.2. The Polynomial Calibration Model

It is necessary to introduce higher-order sensitivities when considering sensitivities which are dependent on the magnetic field itself. This is achieved by replacing the calibration matrix W·S with a matrix polynomial. Therefore, Equation (1) is replaced by

(4) $$B_{\mathrm{cal}}^{T}=R\cdot \sum_{i=1}^{m}H_{i}\left(\begin{array}{c} B_{p,1}^{i}\\ B_{p,2}^{i}\\ B_{p,3}^{i} \end{array}\right),$$

where *m* represents the polynomial degree and $H_{i}$ is the polynomial sensitivity matrix of degree *i*. For *m* = 1 the matrix polynomial becomes linear and equals W·S.

The rotation matrix R, again, is not part of the sensor calibration and, therefore, is separated from the matrix polynomial. It is not possible to determine the rotation matrix with the polynomial model using just one measurement, due to the number of additional matrix coefficients. Hence, R is taken from the linear model. The minimization problem is solved in the same manner as before, now gaining the higher-order sensitivity matrices. In this model, misalignment and sensitivity are combined in the matrix polynomial.

It is necessary to keep the number of parameters in the minimization problem low, in order to not overload the minimization problem and to get satisfactory results. Therefore, it is practical to use different degrees of on-axis coefficients (the diagonal matrix elements) and cross-field coefficients. The on-axis coefficients $o_{i}$, generally, are more important than the cross-field coefficients $c_{i}$, as the latter correct the normally minor misalignment of the sensor’s axes. So, the cross-field degree *n* is mostly chosen to be lower than the on-axis degree *m*. However, *n* should always be greater than zero. Introducing this, Equation (4) becomes

(5) $$B_{\mathrm{cal}}^{T}=R\cdot \left(\sum_{i=1}^{m}O_{i}\left(\begin{array}{c} B_{p,1}^{i}\\ B_{p,2}^{i}\\ B_{p,3}^{i} \end{array}\right)+\sum_{i=1}^{n}C_{i}\left(\begin{array}{c} B_{p,1}^{i}\\ B_{p,2}^{i}\\ B_{p,3}^{i} \end{array}\right)\right),$$

with the on-axis matrices

(6) $$O_{i}=\left(\begin{array}{ccc} o_{11,i} & 0 & 0\\ 0 & o_{22,i} & 0\\ 0 & 0 & o_{33,i} \end{array}\right)$$

and cross-field matrices

(7) $$C_{i}=\left(\begin{array}{ccc} 0 & c_{12,i} & c_{13,i}\\ c_{21,i} & 0 & c_{23,i}\\ c_{31,i} & c_{32,i} & 0 \end{array}\right).$$

### 2.3. Sensors Used in This Study

Three magnetic sensor models from two different manufacturers were studied, namely the HMC5983 by Honeywell and the LSM303DLHC and LIS3MDL by ST Microelectronics. The sensors were chosen based on availability, proposed performance, and preliminary examinations. Five LSM303DLHCs, three HMC5983s, and two LIS3MDLs were examined, to determine the similarity of different sensors of the same model. The AMR sensor chip, being only a few millimeters in diameter, is fixed to an IC-board of approximately 1 × 2 cm in size in all sensor models. The processing electronics and an internal temperature sensor are located on the same die. The supply voltage is in the range of 3–5 V.

The magnetometers share similar characteristics, as they all measure the magnetic field in three orthogonal components and have an operating temperature range of −30/−40 °C to 85 °C [22,23,24]. The linear field range of every sensor model is selectable. In order to achieve the best possible magnetic resolution, the lowest configurable linear field range was selected in all measurements for all sensors; namely ±88 μT for the HMC5983, ±130 μT for the LSM303DLHC, and ±400 μT for the LIS3MDL. The maximum absolute magnetic field of Earth is approximately 60 μT, so any magnetometer’s ability as an electronic compass was not compromised. Fundamental sensor performance parameters, such as magnetic resolution and noise floor, are dependent on the selected linear field range. Thus, the results and conclusions presented in this paper are only valid for the linear field range chosen, too.

As stated by the manufacturer, the magnetic resolution varies from 14.6–90.9 nT and the noise standard deviation from 200–410 nT; although, the LSM303DLHC’s data sheet did not give details about the latter. Axial misalignment was only given for the HMC5983, with 1° to 2°. The maximum data output rate lies between 80–220 Hz, respectively. For better comparability, sampling frequencies of 80 Hz for the LIS3MDL and 75 Hz for the other two sensor models were chosen. The sampling frequencies could not be chosen identically, due to the internal frequency generation by the fixed clock dividers.

The HMC5983, additionally, had a sensitivity temperature compensation which maintained the sensor sensitivity in the operating temperature range. The LSM303DLHC had an on-board accelerometer, which was not needed for the calibration and was, therefore, deactivated.

### 2.4. Calibration Measurements and Analysis

**Figure 2:**
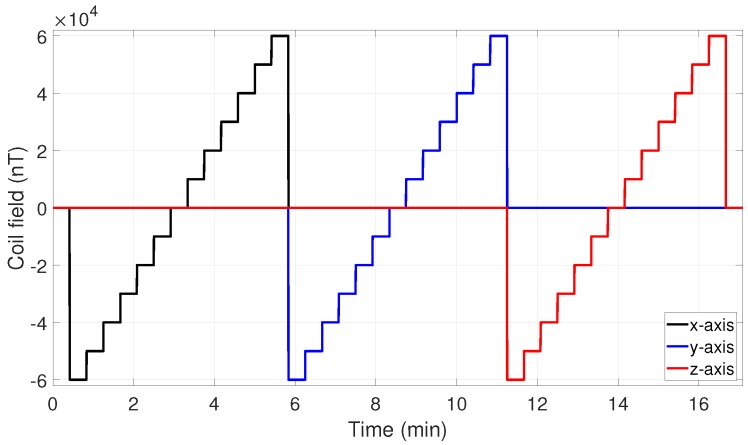
Applied coil fields of the LIN-60000 measurement.

All measurement data were analyzed with own MATLAB code (MATLAB version 2018a). Measurement data, protocol and code are available online under doi 10.5281/zenodo.2591195 as Supplementary Material. There were five different types of measurements performed, four of them in the Magnetsrode calibration facility. The most important one is the linearity measurement, with which, using the two calibration models, the sensitivities, misalignment angles, offsets, and field dependencies in static fields can be determined. The performed field program LIN-60000 is shown in Figure 2. It applies constant fields for 25 s and was used on every magnetometer.

A single offset and residual coil field measurement was performed beforehand, to ensure a negligible influence of the coil system on the measurements. The sensor was measured at null field and turned by 180° around each main axis. The sensor offset can be expressed by

(8) $$B_{\mathrm{off}}=\frac{B_{\mathrm{nor}}+B_{\mathrm{tur}}}{2},$$

while the residual field of the coil system is

(9) $$B_{\mathrm{res},\mathrm{coil}}=\frac{B_{\mathrm{nor}}-B_{\mathrm{tur}}}{2}.$$

$B_{\mathrm{nor}}$ is the field at normal sensor orientation, while $B_{\mathrm{tur}}$ is the field with the sensor orientation turned by 180°. The measurement showed a residual coil field of 2.5 nT maximum. The effect of the inaccuracy of the coil can be neglected because of the sensor resolutions of 14–90 nT. As a result, the sensor offset does not need to be compensated for the difference between the normal and turned field and, therefore, can be determined by just taking the sensor output at null field.

**Figure 3:**
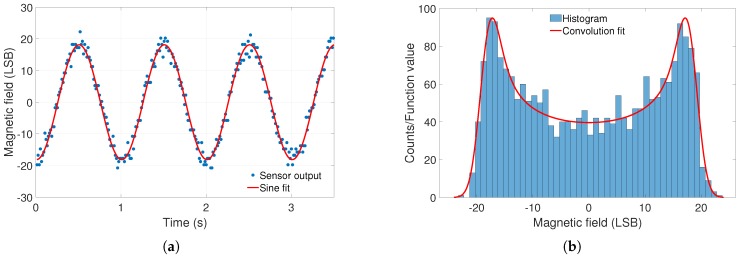
Fitting for determination of the frequency dependency of the sensitivity. (**a**) An excerpt of a sinusoidal fit to the LSM #1 data at 1 Hz, and (**b**) the corresponding histogram and fitted density function.

As the LIN-60000 measurement does not give information about frequency and temperature dependencies, these measurements had to be done separately. The former was determined by applying a sinusoidal field with a peak-to-peak amplitude of 4000 nT in one field direction. The sensor was slanted, so the applied field covered all three axial components. The frequency was changed discretely between values of 1, 3.7, 10, 25, 37, and 61 Hz. The change in sensitivity can be acquired by determining the sine amplitude for each frequency. High noise and low resolution prevent a good fitting at all sine frequencies, except for 1 Hz and, partly, 3.7 Hz. An exemplary fit is shown in Figure 3a.

Therefore, another evaluation technique had to be used to determine the sine’s amplitude and acquire comparable results for different frequencies. Instead of direct analysis of the sensor output, a density function was fitted to the histogram data of the sensor with the following mathematical background. The density of a sine $P_{S}$ has a characteristic shape, with maxima at the positive and negative amplitude, and is expressed by

(10) $$P_{S}\left(y\right)=\frac{1}{\pi \sqrt{A^{2}-y^{2}}},$$

where *A* is the amplitude of the sine [25]. The sensors show Gaussian noise, so the sine density function has to be convoluted with

(11) $$P_{G}\left(x\right)=\frac{1}{\sqrt{2\pi }\sigma }\exp\left(-\frac{x^{2}}{2\sigma^{2}}\right),$$

the symmetric Gaussian noise density, where σ is the standard deviation of the sensor output at zero field. The Gaussian noise density can be determined and the convolution is fitted to the histogram data of the sensor. The sine amplitude can then be calculated with the convolution. An example of this is shown in Figure 3b. There is a determination error of the amplitude caused by sensor noise and fitting inaccuracy. In the following, this error is neglected, because it is rather low and is further reduced by taking the mean of several measurements of the same sensor. Both fits shown in Figure 3 are determined by minimizing the variance of the difference between the fitted function and the data.

The temperature behavior of the sensors was examined by a sequential measurement. At first, the sensor within the thermally isolating box was heated up to 70 °C. Then, the LIN-60000 program was repeated while the temperature slowly decreased to room temperature. Due to the long time of the measurement (it takes the box several days to cool down), it was assured that there were no big temperature gradients within the box affecting the measurement. Therefore, the temperature can be assumed to remain constant during a LIN-60000 program, as the program time is short compared to the cooling duration.

**Figure 4:**
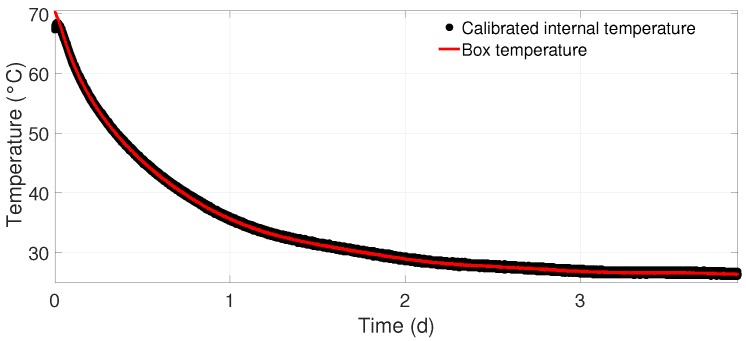
Temperature calibration curve of the internal temperature sensor for the LIS3MDL #2.

The temperature was measured with sensors inside the box and, if existent, an internal temperature sensor on the IC. The latter was calibrated with the temperature data from the box and then used for the determination of temperature effects on the sensor’s measurement capabilities. An example for such a calibration is shown in Figure 4.

The internal temperature data fit the box temperatures very well in all calibrations. Only at the beginning of the measurement there were deviations of the temperature data, which can be attributed to differing heat transfer behavior of the air inside the box and the AMR sensor material.

Finally, noise determination was performed by placing the magnetometers inside a three-layer mu-metal shielded container, preventing the influence of external magnetic fields. The measurements lasted five minutes. Two sensors were measured in parallel, only about 1 cm apart from each other. It was assured that the sensor’s electronics do not create magnetic fields that could disturb the measurement of the adjoining sensor by comparing dual and single sensor measurements. The power spectral density of the magnetometers could be estimated from the noise measurements.

## 3. Results

Many different parameters are important for a complete magnetometer characterization. An overview of the parameters obtained in this study for the different sensor models is shown in Table 1.

### 3.1. Sensitivity

Sensitivity, one of the most important calibration parameters, was found to be best in the LIS3MDL sensor. It is characterized by similar linear sensitivities for all axes of the two sensors of this type examined. The linear sensitivities only deviated minimally from the value given in the data sheet. Contrastingly, the sensitivity was found to be quite scattered around, and even away from the reference value, for the other two sensor models. In addition, a difference between the magnetometer’s axes could be observed for those sensors. The z-axis sensitivity was significantly higher than those of the x- and y-axes. This might be caused by the production process of the sensor (see, for example, [26] (pp. 115–132)). We assume that two of the sensor axes (in this case, x and y) were placed on one wafer, while the third axis had to be vertical. The attachment of this third Wheatstone bridge might cause difficulties, as orthogonality has to be assured. Therefore, the active surface is reduced, resulting in a lower resolution. As detailed information about the production processes of the examined sensors were not available at the time of publication, we can only suspect this to be the reason for the differences in sensitivity for the HMC5983 and LSM303DLHC.

**Figure 5:**
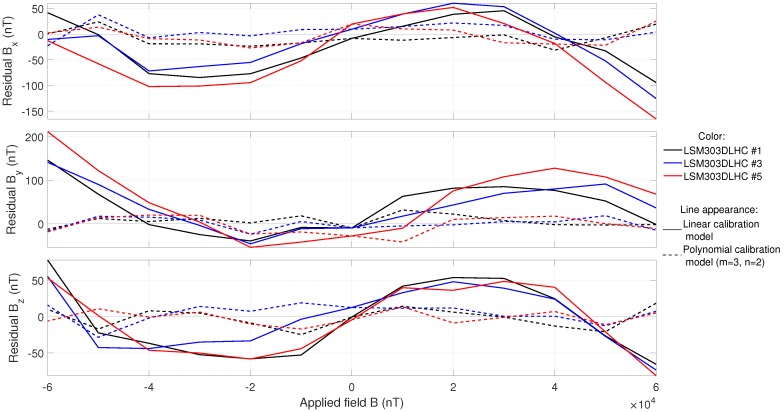
Comparison of on-axis residual fields in coil coordinates, for the two different calibration models, for three of the five LSM303DLHC sensors.

The sensitivities reported so far are linear sensitivities, that were calculated with the linear calibration model. The polynomial calibration model, however, gave sensitivity polynomials for each matrix element. The first order of the on-axis elements only differed slightly from the linear sensitivities. Nevertheless, differences could be seen in the residuals. The polynomial model significantly lowered the overall residual norm of the fitted data and the known field configuration. This suggests a better fit. Large improvements were observed, especially with higher on-axis polynomials. This effect can be attributed to the course of the on-axis residuals when plotted over the applied field (see Figure 5), approximated by third-order polynomials for the LSM303DLHC and HMC5983. As a result, the on-axis polynomials of order 3 lowered the overall residuals by up to 80%. Examples of these improvements are shown in Figure 5.

The cross-field coefficients had a smaller influence, but they were capable of reducing the overall residuals by additional 10%. Altogether, the polynomial calibration model largely improved the quality of the calibration. For the LSM303DLHC and HMC5983 sensors, it was sufficient to use higher-order polynomials, of order *m* = 3 and *n* = 2 (see Equation (5)). The LIS3MDL showed no collective or predictable behavior and, thus, the use of the polynomial calibration model was less efficient. However, it also significantly reduced the overall residuals by about 50% for adequate orders of *m* = 2 and *n* = 1.

**Figure 6:**
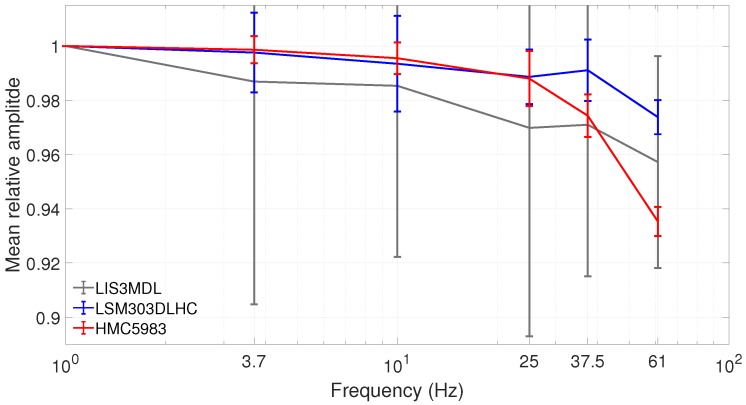
Mean relative amplitudes for all three sensor models, with standard deviation error depicted by error bars. All amplitudes are normalized with the value at 1 Hz.

As stated above, knowledge of the sensitivity temperature and frequency dependency is vital for a complete calibration. The examination of the frequency behavior showed a decrease of amplitude response in the sensors with higher frequencies, as shown in Figure 6.

As sensitivity and amplitude response are reciprocal to each other, the sensitivity value increases with frequency, which is synonymous with poorer resolution. The amplitude response lay in the range of 90–97% of the 1 Hz value at 61 Hz. We strongly suspect the bandwidth of the sensor caused the decrease in amplitude response. Unfortunately, the manufacturer data sheets did not provide information about sensor bandwidth; however, our measurement data indicated that the 3dB-bandwidth of all three sensor models was considerably higher than the examined frequency range, which was bordered by the sampling rate of the sensors. The 3dB-bandwidth was also higher than in other magnetometers for space applications. For example, the THEMIS fluxgate magnetometers showed relative amplitudes of 70% at 60 Hz [27], which corresponds to a 3dB-bandwidth of 60 Hz. Therefore, the AMR magnetometers are superior in measuring high-frequency fields, which agrees with earlier findings, such as in [10].

To compare the sensor models, the mean relative amplitude response is shown, in Figure 6, for each sensor model separately. There, the amplitude response was normalized with the 1 Hz value and the mean of all measured axes in one sensor model was taken for each frequency separately. The error bars depict the standard deviation error of the mean. For the LSM303DLHC, and especially the HMC5983, these errors were lower than the overall change in relative amplitude. Thus, the decrease in amplitude can be trusted. This did not apply for the LIS3MDL. In this sensor model, the decrease could only be suspected to be due to the large errors.

**Figure 7:**
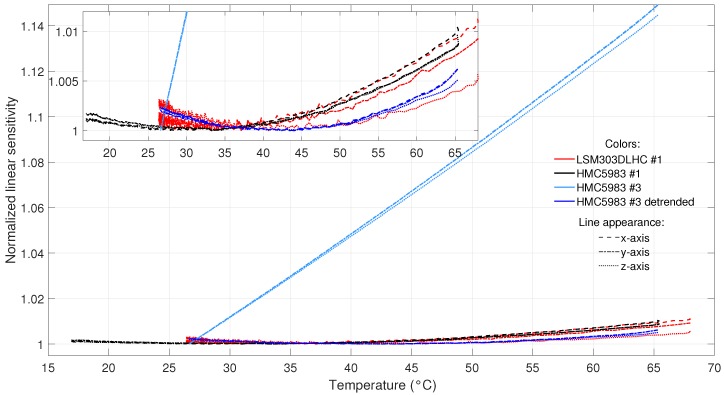
Sensitivity temperature dependency for the LSM303DLHC #1 and HMC5983 #1 and #3. The different sensors are represented by different colors, and the x-, y-, and z-axes by dashed, dashed-dotted, and dotted lines, respectively. The HMC5983 #3 had no internal temperature compensation and shows a linear dependency (light blue lines). For this sensor, additional courses (dark blue lines) with the linear trend subtracted (‘detrended’) are also shown. The remaining sensitivity course can be approximated by a second-order polynomial as in the other shown sensors, which is magnified in the inlay.

The temperature measurement evaluation led to some interesting findings. All of the temperature-compensated HMC5983 and LSM303DLHC sensors showed a weak quadratic temperature dependency of the sensitivity. For the HMC5983 #3 with a non-functional internal temperature sensor, a stronger linear dependency was found. Both cases are depicted in Figure 7.

As the HMC5983 sensor model is known to be temperature-compensated, this can be suspected for the LSM303DLHC sensor model too, due to its similar behavior (there was no information in the LSM303DLHC data sheet regarding temperature compensation). It can be assumed that the temperature compensation subtracts the linear dependency, leaving weak higher-order dependencies. This is conformal with earlier findings on the temperature behavior of thin-film AMR sensors in [26]. The sensitivity $S_{a}$ of an analog AMR magnetometer can be expressed by

(12) $$S_{a}∼\frac{H_{x}}{U_{\mathrm{out}}}=\frac{\rho }{\Delta \rho }\frac{H_{k}}{U_{0}}$$

(derived from [10,26] (pp. 17–22)), where $H_{x}$ is the magnetic field, $U_{\mathrm{out}}$ and $U_{0}$ are the output and input voltage, $H_{k}$ is an anisotropic field constant, and ρ and Δρ are the resistivity at zero field and resistivity change at the field $H_{x}$, respectively. The last three parameters change linearly with temperature [26] (pp. 138–139). As a result, the sensitivity $S_{a}$ is proportional to the temperature. Temperature compensation makes use of this linearity.

To compensate for the remaining second-order dependency, the second-order polynomial relationship between sensitivity and temperature had to be calculated. For the HMC5983 and LSM303DLHC sensors, all coefficients of determination were above 0.94, showing a good calibration. The drift can be determined by the differentiation of the sensitivity polynomial with respect to temperature. Assuming a non-changing relation, the resulting drifts range from −150 to 100 pT/LSB/K for the LSM303DLHC and HMC5983. Sensitivity drifts are lowest and highest at the operating temperature boundaries of −40 and 85 °C, respectively. The conversion occurs in the range of 20 to 50 °C. Without compensation, the drift went up to 340 pT/LSB/K for the HMC5983 #3. LIS3MDL sensors showed major fluctuations of sensitivity in the measured temperature range, most certainly caused by high noise. Drift values were negligible, with a range of −5 to 11 pT/LSB/K for this sensor model.

The second-order compensation seems superfluous, considering the low sensitivity drifts. However, the sensitivity change amounts for up to 1% in low temperatures. This means an error of 300 nT for a field of 30,000 nT (reference value of the absolute magnetic field at the equator [28]), which should be taken into account.

### 3.2. Offset

As stated in the data sheets for the HMC5983 and LIS3MDL, both sensor models are offset-compensated, so the maximum field range is not reduced. Such a compensation can be suspected in the LSM303DLHC, as well, because of its similarity to the HMC5983 and LSM303DLHC measurement data. Offset compensation is carried out internally, leaving all measured sensors with significant offsets of up to 53,700 nT (see Table 1). The offsets are constant on a short time-scale. It is inevitable to compensate for the offsets, as they are on the order of the Earth’s magnetic field and the sensor measurement range. This has to be done prior to use, or in-situ, for every single sensor individually, as the offsets seem to be randomly distributed. The LIS3MDL showed the highest offsets, while the other two sensor models had comparably lower ones.

The offset changes with the temperature, which can be described by a second order polynomial (similar to the sensitivity temperature dependence). However, a linear calibration was sufficient, with nearly all coefficients of determination over 0.98. So, offset change with temperature was determined using the linear fits. The changes were both positive and negative, with no paramount rule; the axes and sensors showed different offsets and offset changes within sensor-specific boundaries. Looking at the offset change, the LIS3MDL, again, had the highest absolute values, with up to 598 nT/K; while the HMC5983 and the LSM303DLHC showed maximum values of 57 and 30 nT/K, respectively.

Long-term offset alteration was observed, although the reason could not be determined. Most likely, mechanical stress or a permanent temperature effect caused by heating and cooling was the cause. Low magnetic fields can be ruled out, because the offset was stable in all linearity and frequency measurements.

### 3.3. Noise

**Figure 8:**
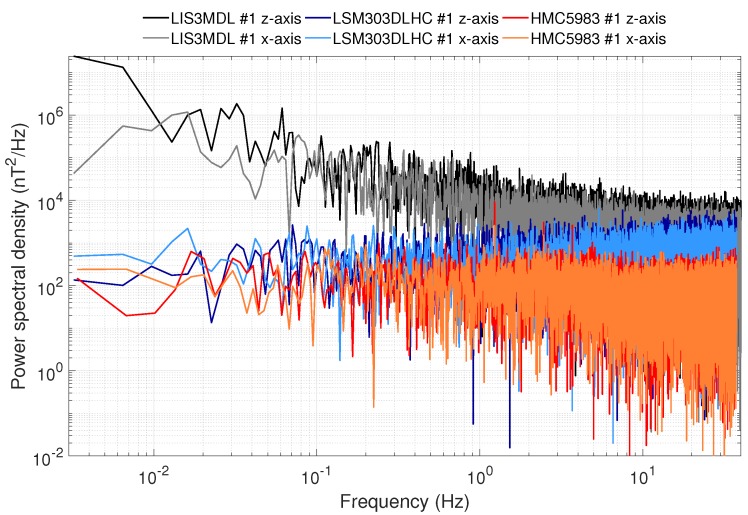
Exemplary noise spectral densities of all three sensor models.

Sensor noise had the most severe impact on the measurements. As depicted in Table 1, the noise standard deviation was way higher than the resolution, leading to a low signal-to-noise ratio. This significantly reduced the ability of the sensors to measure low fields, in the range of a few hundred nT. The LIS3MDL sensors, especially, showed extremely large noise. This can be seen when looking at the typical noise spectral densities (NSD) of all three sensor models, as shown in Figure 8.

The HMC5983 had the lowest noise, followed by the LSM303DLHC. As the calculated standard deviations suggest, the z-axis had higher noise than the x- and y-axes. This effect can likely be attributed to the production process, similar to the axial differences in the sensitivity values. The HMC5983 showed a noise standard deviation lower than its sensitivity. At first sight, this seems crude, but it is possible, as magnetometer value fluctuations are very low and, thus, the output values are mostly the same. The standard deviation is calculated with the common estimator, resulting in the low standard deviation values for the HMC5983. Figure 8 also shows the major difference between the LIS3MDL and the two other sensor models. The latter sensors showed a near-constant NSD over all frequencies (white noise), while the former had a linearly-decreasing NSD in double-logarithmic representation, and so showed 1/f noise. This can also be observed by looking at the raw noise data, where low frequency output changes occur for the LIS3MDL, but not for both of the other sensors.

### 3.4. Misalignment

Here, internal misalignment is defined as the deviation from orthogonality between the magnetometer axes. It was comparable in all sensors, except the y-z angle of the HMC5983. This angle was around 82.5°, and so the misalignment was about 7.5°. Again, this suggests difficulties in the installation of the third axis in three-dimensional AMR magnetometers. All other misalignment angles rarely exceeded 2.0°. There was no structure discernible in the internal misalignment, and it can be corrected by precise calibration.

Additionally, the sensors showed alignment uncertainty induced by noise. Precise orientation determination can be compromised by the high noise, as the lengths of the axial vectors are altered by it. As a result, the orientation of the measured field vector deviates slightly from the actual orientation. Taking the trigonometric relations into account, the maximum noise-induced misalignment angle between the two field vectors $\xi_{\max}$ with the given axial noise $N_{\mathrm{xy}}$, and $N_{z}$ can be expressed by

(13) $$\xi_{\max}=\arctan\left(\frac{\sqrt{N_{\mathrm{xy}}^{2}+N_{z}^{2}}}{\left|B_{\mathrm{EMF}}\right|-N_{\mathrm{xy}}}\right),$$

where $B_{\mathrm{EMF}}$ is the magnetic field vector of the Earth. Using Equation (13) with an equatorial absolute reference value at zero altitude, $B_{\mathrm{EMF}}$ = 30,000 nT, and the mean standard deviation noises from Table 1, the noise-induced misalignment angle amounts to $\xi_{\max}$ ≈ 0.38° for the LSM303DLHC, $\xi_{\max}$ ≈ 0.15° for the HMC5983, and $\xi_{\max}$ ≈ 1.5° for the LIS3MDL. The noise-induced misalignment cannot be reduced by calibration but, instead, by taking the mean value of several measured field vectors. Although the calculated values are mostly lower than the internal misalignment, for higher altitudes $B_{\mathrm{EMF}}$ becomes smaller and, thus, $\xi_{\max}$ becomes greater. As an example, at an altitude of 800 km, a common altitude for Earth-observation satellites, the minimum absolute field is $B_{\mathrm{EMF}}$ = 16,000 nT [28]. As a result, the noise-induced misalignment is more than twice as big. It also increases when taking into account the maximum noise, as opposed to the standard deviation noise. This enlarges the noise-induced misalignment significantly, but is exaggerated when looking at overall mean alignment uncertainties.

## 4. Discussion

It was possible to determine a complete calibration for the measured AMR sensors HMC5983, LSM303DLHC, and LIS3MDL. The capabilities and success of the different measurements and evaluation techniques could be verified. Various measurement parameters for all three examined sensor models could be determined. Most measurement parameters differed quite largely from the information given in the manufacturer’s sensor data sheets. Other characteristics and parameters not considered in the data sheets could be evaluated in this study. The gathered information indicated that a calibration is indispensable for the use of the sensors in high-precision attitude determination in sophisticated fields of application, such as small satellites or navigational electronics. If the sensors are used in special applications with specific calibration parameters being absent, the corresponding calibration measurements can be dropped. For example, frequency dependency determination is unnecessary when measurements are only done in static fields.

Some of the examined parameters showed low deviations across different sensors of the same model. For example, the linear sensitivities were quite similar, with deviations mostly below ±5%. For the HMC5983 and LSM303LHC, the z-axis showed the worst sensitivity; meanwhile, the sensitivities of the LIS3MDL were almost the same for all axes. The noise characteristics were very similar across different sensors of the same model, as well. Noise spectral density, noise standard deviation, and, therefore, alignment uncertainties induced by noise only differed by a few percent at most. Noise can be reduced by down-sampling and filtering. All of these small-deviation parameters can be taken from this paper, in order to avoid calibration.

The other parameters are individual for each sensor, as they show large deviations across sensors of the same model and, thus, have to be calibrated prior to sensor use. Values presented in this paper can only be taken for error estimations. Such parameters are offset and offset change with temperature, as well as misalignment of the sensor axes towards each other. The latter showed the highest misalignment towards the z-axis.

All sensors are temperature-compensated, by means of an internal temperature-sensor, leaving nearly negligible changes of sensitivity with temperature. In contrast, offset changes significantly with temperature. Both parameter’s dependence on temperature can be expressed by a low-order polynomial. The sensitivity decrease with higher frequencies was less than 10% at 61 Hz.

The LIS3MDL had, by far, the lowest (and therefore best) sensitivity of all sensors; however, this was dominated by high noise. The sensor had the highest noise of all three, by far. This significantly reduced the sensor’s capabilities in every aspect. Calibrations were compromised in most cases; for example, frequency and temperature dependencies of the sensitivity could only be determined with very high errors. A systematic field-dependent sensitivity behavior could not be determined, as opposed to the other two sensor models. The internal misalignment of the LIS3MDL was as low as in the LSM303DLHC, but the alignment uncertainty induced by noise hugely diminished the sensor’s orientation determination capabilities. In general, the LIS3MDL showed the worst overall performance of all three sensor models.

The LSM303DLHC and the HMC5983 were quite similar. Both sensors showed third-order sensitivity dependency, regarding the magnetic field. The introduced polynomial calibration model could be used to reduce the residuals by up to 90%, compared to the linear calibration model. The HMC5983 had slightly better sensitivity and noise values and, therefore, can measure smaller fields with higher precision. Maximum internal misalignment is very high in the HMC5983. This can be compensated for by using calibration, only leaving noise-induced misalignment which is, again, lower in the HMC5983. Sensitivity temperature and frequency dependency were comparable. High frequencies reduced the sensitivity by up to 7%, an effect which was stronger in the HMC5983. Both sensors showed a similar maximum offset and offset migration with temperature.

As a result, the HMC5983 seems to be the best sensor model of the three, especially in orientation determination and field precision. However, the HMC5983 sensors frequently showed extreme outliers, while the LSM303DLHC sensors had very few. These outliers can alter the signal, even if using moving-mean techniques combined with live processing. Nevertheless, the number of examined magnetometers was not high enough to confirm a systematic outlier sensor characteristic in the HMC5983. The LSM303DLHC is the best choice for future projects, as the HMC5983 was discontinued by the manufacturer at the date of publication. Other sensor models were not examined, due to worse performance either specified by the manufacturer or observed in preliminary basic sensor examinations.

With the parameters, calibration techniques, and sensor characteristics shown in this publication, operators will be able to chose the right sensor for their application, use the given measurement parameters to partially avoid complex and time-intensive calibration for low-deviation parameters, and perform error estimations for the remaining ones. Operators with a capable calibration facility can use the calibration techniques introduced above, which are specifically geared towards digital AMR magnetometers.

## Figures and Tables

**Table 1:** Important calibration parameters of the three sensor models. Range values take into account all sensors of the same model and show the maximum value spread.

		LSM303DLHC	HMC5983	LIS3MDL
Linear sensitivity range (nT/LSB)	x/y-axis	78.6 ± 2.6	65.7 ± 2.5	14.7 ± 0.3
	z-axis	93.9 ± 2.1	73.0 ± 4.8	14.9 ± 0.0
Misalignment angle	median	0°41${}^{\prime }$ (0.68°)	1°11${}^{\prime }$ (1.18°)	1°00${}^{\prime }$ (1.00°)
	maximum	2°18${}^{\prime }$ (2.30°)	7°37${}^{\prime }$ (7.62°)	3°25${}^{\prime }$ (3.42°)
Alignment uncertainty induced by noise	for $\left|B\right|$ = 30,000 nT	0°23${}^{\prime }$ (0.38°)	0°09${}^{\prime }$ (0.15°)	1°30${}^{\prime }$ (1.51°)
	for $\left|B\right|$ = 16,000 nT	0°43${}^{\prime }$ (0.72°)	0°17${}^{\prime }$ (0.29°)	2°51${}^{\prime }$ (2.85°)
Mean noise standard deviation (nT)	x/y-axis	133.7	54.3	331.3
	z-axis	147.1	59.5	706.3
Mean noise density at 1 Hz $\left(\mathrm{nT}/\sqrt{\mathrm{Hz}}\right)$	x/y-axis	21.2	11.5	86.6
	z-axis	23.4	11.9	124.2
Offset (nT)	minimum	−2.50 $\times \;10^{4}$	−2.55 $\times \;10^{4}$	−5.05 $\times \;10^{4}$
	maximum	2.09 $\times \;10^{4}$	9.9 $\times \;10^{3}$	5.37 $\times \;10^{4}$
Linear sensitivity temperature drift (pT/LSB/K)	calculated range at 25 °C	−15.3 ± 25.0	−5.6 ± 1.0	2.6 ± 2.4
	calculated mean formula	1.4 × T(°C) − 53	1.0 × T(°C) − 30	0.01 × T(°C) + 3
Offset temperature drift (nT/K)	range	15.2 ± 14.3	12.5 ± 44.4	−188.2 ± 410.4
	maximum absolute value	29.5	56.8	598.6

**Figure A1:**
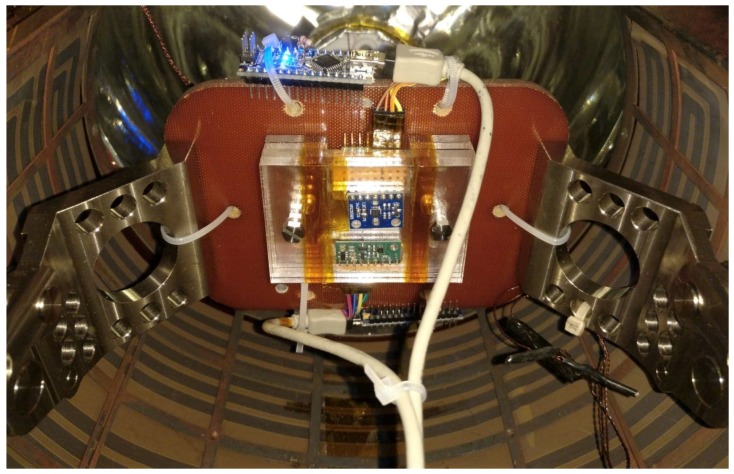
Detailed inside view of the measurement box (magnification of Figure A2a). The two sensors in the middle of the picture are being tested. Data transfer and current supply are provided by USB cables; primary data processing is taking place in the arduino boards placed on top and at the bottom of the holding plate. Downwards and right of the holding plate, three wires with black tape on their ends can be seen; those are three of the eight temperature sensors inside the measurement box.

**Figure A2:**
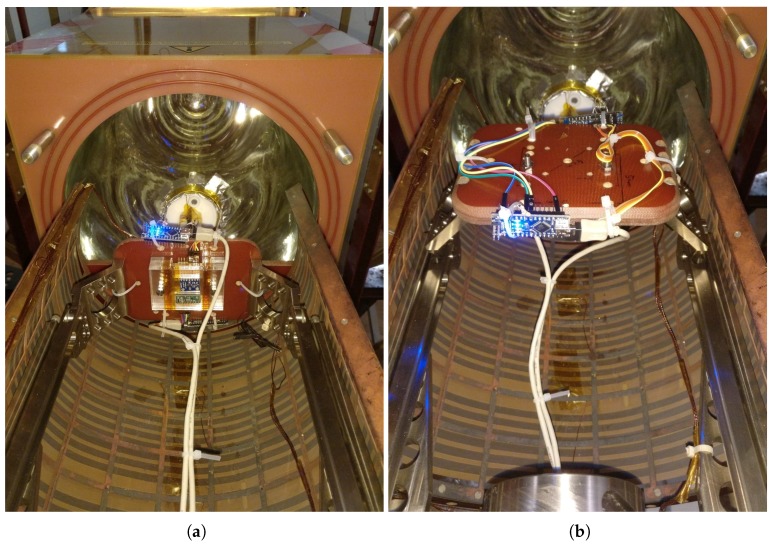
Inside view of the measurement box (the yellow box in Figure 1). The underlying grid forming a semicircle inside the box is the heating system (here, the whole structure is pulled out of the box). The sensors being tested are fixed on the transparent plastic structure (see the middle of (**a**)), which is screwed to the brown holding plate. This plate can be turned, as is shown in (**b**). All materials inside and outside the box are non-magnetic.

## Supplementary Materials

The following are available online at http://doi.org/10.5281/zenodo.2591195. Folder “Data”: Measurement data in form of .txt files, calibration profiles, and temperature data; Folder “MATLAB Code”: MATLAB calibration code (MATLAB version 2018a); File “Measurement_Log.xlsx”: complete log-file of measurements with additional information.
